# Nitric Oxide Is Essential for Melatonin to Enhance Nitrate Tolerance of Cucumber Seedlings

**DOI:** 10.3390/molecules27185806

**Published:** 2022-09-07

**Authors:** Yiting Zhang, Ailong Liu, Yanwei Hao, Wei Su, Guangwen Sun, Shiwei Song, Houcheng Liu, Riyuan Chen

**Affiliations:** College of Horticulture, South China Agricultural University, Guangzhou 510642, China

**Keywords:** melatonin, nitric oxide, nitrate stress, gene expression, root growth, cucumber seedlings

## Abstract

Melatonin (MT) and nitric oxide (NO) in plants can function cooperatively to alleviate salt stress, sodic alkaline stress and immune response, as well as adventitious root formation. The interaction of MT and NO on the nitrate stress tolerance of cucumber seedlings are not well understood. We investigated the effects of exogenous MT, NO donor (SNP) and NO scavenger (cPTIO) on the growth; photosynthesis; characteristics of root morphological; accumulation of mineral elements, endogenous NO, MT, IAA and ABA; and related genes expression in cucumber (*Cucumis sativus* L. “Jin You No. 1”) seedlings grown under high nitrate condition (HN). The results showed that MT and NO independently alleviated the inhibition of growth and photosynthesis capacity of cucumber seedlings under nitrate stress. NO was required for MT to enhance the root activity, root length, lateral root number and the accumulation of calcium, magnesium and iron in the roots of cucumber seedlings grown under nitrate stress. Consistently, the expression of adventitious rootless 1 gene (*CsARL1*) was modulated. Furthermore, exogenous MT induced accumulation of endogenous MT, NO, indole-3-acetic acid (IAA) and abscisic acid (ABA), mainly within 24 h after treatment, in which MT and NO were further increased at 48 h and 96 h, IAA and ABA were further increased at 16 h in the presence of SNP. In contrast, the accumulation of endogenous IAA, MT and ABA slightly decreased within 24 h, NO significantly decreased at 192 h in the presence of cPTIO. Correspondingly, the expression levels of genes involved in nitrogen metabolism (*CsNR1* and *CsNR2*), MT metabolism (*CsT5H*, *CsSNAT2* and *Cs2-ODD33*), auxin carriers and response factors (*CsAUX1*, *CsGH3.5*, *CsARF17*), ABA synthesis and catabolism (*CsNCED1*, *CsNCED3* and *CsCYP707A1*) were upregulated by MT, in which *CsNR1*, *CsNR2*, *CsAUX1*, *CsNCED3* and *CsT5H* were further induced in the presence of SNP in roots of cucumber seedlings. These observations indicated that NO act as a crucial factor in MT, alleviating nitrate stress through regulating the mechanism of root growth in cucumber seedlings.

## 1. Introduction

Nitrogen (N) is one of the important nutrients for plant growth and development [1]. Application rates for N fertilizers have increased dramatically in intensive agricultural systems, especially those used for protected vegetable production in China [2,3]. Excessive use of nitrogen fertilizers in greenhouses has resulted in accumulation of NO_3_^−^ and secondary soil salinization leads to reduced yield and quality of vegetables [4,5]. The use of exogenous substances may be an alternative means to solve this problem.

Melatonin (MT, *N*-acetyl-5-methoxytryptamine) is a pleiotropic, orchestrating regulator, existing in almost all organisms [6]. The MT was synthesized from tryptophan in four sequential reactions catalyzed by tryptophan decarboxylare (TDC) [7], tryptamine 5-hydroxylase (T5H) [8], serotonin *N*-acetyltransferase (SNAT) [9,10] and *N*-acetylserotonin methyltransferase (ASMT) [11,12,13]. Four independent genes belonging to the 2-oxoglutaratedependent dioxygenase (2-ODD) family, 2-ODD11, 2-ODD19, 2-ODD21, and 2-ODD33, encode enzymes that catalyze the conversion of melatonin to 2-hydroxymelatonin [14]. The MT plays a vital role in root development [15], photosynthesis [15,16], leaf senescence [17], seed germination [18] and circadian rhythms [19]. As a potent, naturally occurring antioxidant, MT firstly serves as the first line of defense against environmental oxidative stressors [20]. MT alleviates the inhibitory effects of NaCl stress on germination, mainly by regulating the biosynthesis and catabolism of ABA and GA_4_ in cucumber [18]. The pretreatment with MT reduced the oxidative damage under NaCl stress by scavenging directly H_2_O_2_ or enhancing activity of antioxidant enzymes and concentrations of antioxidants in cucumber seedlings [21]. MT mediates the regulation of ABA metabolism, free-radical scavenging, and stomatal behavior in two *Malus* species under drought stress [22]. MT promotes water stress tolerance, lateral root formation and seed germination in cucumber [23]. MT enhanced tomato tolerance to alkaline stress by maintaining ion homeostasis [24], and NO acts as a down-stream signal, which is involved in the MT-induced tomato tolerance to alkaline stress [25].

Cucumber (*Cucumis sativus* L.) is one of the primary greenhouse vegetables in China and is very sensitive to nitrate stress [26]. In recent years, cucumber production was seriously restricted by nitrate stress in protected facilities in China [4,21]. Excessive NO_3_^−^ levels in the soil severely inhibit the growth and development of cucumber, showed shorter and narrower stems and weaker root systems [5]. Therefore, enhancing the nitrate tolerance of cucumber is important for practice production in China. Exogenous MT enhanced nitrate tolerance by increasing the activity of antioxidant enzymes and antioxidants content in tomatoes [27] or cucumber seedlings [16]. MT improved the growth of alfalfa by modulating the morphology, mineral nutrition, nitrogen metabolism and energy status under nitrate stress [28]. Exogenous MT enhanced the capacity for nitrogen metabolism and uptake of mineral elements, as well as modulating the activities of enzymes involved in nitrate metabolism and the expression of their related genes (*CsNR* and *CsGOGAT*) in cucumber seedlings under nitrate stress [29]. These observations indicated MT positively influences cucumber seedlings’ responses to nitrate stress by regulating nitrogen metabolism and the antioxidant system.

It was demonstrated that MT promoted adventitious root or lateral root development by regulating IAA and nitric oxide (NO) signaling in tomatoes [30] or *Arabidopsis thaliana* [31]. MT triggers NO accumulation by the upregulating of *SlNR* expression and down-regulation of *S*-nitrosoglutathione reductase (*GSNOR*) expression, and NO increased IAA accumulation in tomatoes [30]. NO was involved in the ethylene-induced adventitious root development in cucumber explants [32]. The RNA-seq research indicated that MT promoted lateral root formation of cucumber by activating root-related hormones and transcription factor pathways [33]. Much of these effects are ascribed to the action of NO as a down-stream signal molecule in the MT-regulating root growth pathway.

Previous research showed that NO contributed to the MT-enhanced salt stress, sodic alkaline stress, immune response, adventitious root formation, etc. However, the effects of interaction of MT and NO on the nitrate stress tolerance of cucumber seedlings are not well understood. In the present study, we investigated the effects of MT and NO on the photosynthesis capacity; pigment content; root morphological characteristics; accumulation of mineral elements; changes of endogenous NO, MT, IAA and ABA; and on related gene expression of cucumber seedlings grown under nitrate stress. The outcome of this research should help gain a deeper insight into the interaction of MT and NO in regulating the nitrate stress tolerance.

## 2. Results

### 2.1. Experiment I: External MT and NO Effect on the Growth of Cucumber Seedlings under Nitrate Stress

**Figure 1:**
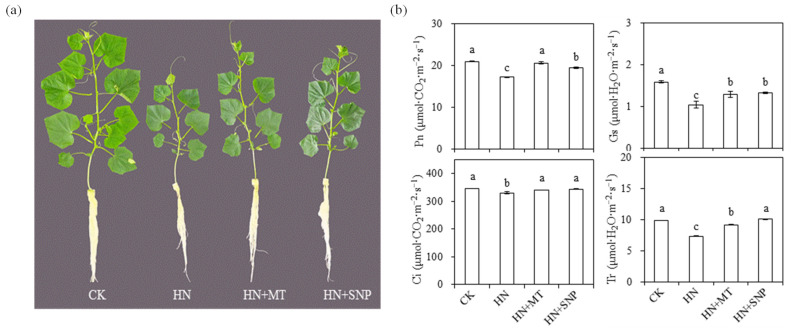
Effects of melatonin and nitrate oxide on the appearance of cucumber seedlings and photosynthesis capacity. (**a**) Appearance of seedlings. (**b**) Photosynthesis capacity, including Pn, net photosynthetic rates. Gs, stomatal conductance. Ci, intercellular CO_2_ concentration. Tr, transpiration rate. Error bars represent standard deviations of the means of three independent replicates. Different letters indicate significant differences between treatments by Duncan’s multiple range test (*p* < 0.05).

In order to assess the effect of exogenous MT and NO in the alleviation of high NO_3_^−^ stress cucumber seedlings, NO_3_^−^ concentration in the nutrient solution was elevated up to 75 mM, and 2 μM MT and 100 μM SNP (NO donor) were applied based on high NO_3_^−^, respectively. As shown in Table 1 and Figure 1a, the appearance of seedlings and growth parameters were significantly restricted under HN treatment, compared with CK. The addition of MT and NO alleviated the growth inhibition, in which dry weight was increased to the same level as CK. The addition of MT and NO also alleviated the reduction in photosynthetic capacity under nitrate stress (Figure 1b). Compared with HN, the Pn and Ci in HN + MT treatment and Ci and Tr in HN + SNP treatment were significantly enhanced (Figure 1b).

**Figure 2:**
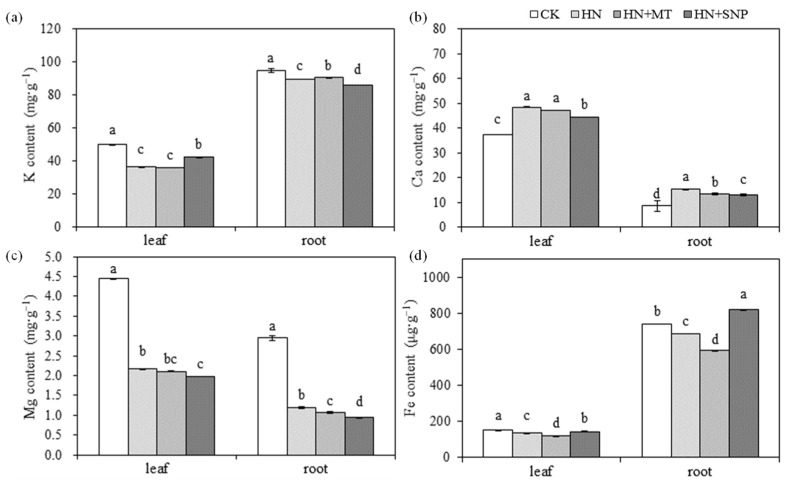
Effects of MT and NO on mineral elements content of cucumber seedlings under nitrate stress. (**a**) Potassium (K). (**b**) Calcium (Ca). (**c**) Magnesium (Mg). (**d**) Iron (Fe). Error bars represent standard deviations of the means of three independent replicates. Different letters indicate significant differences between treatments by Duncan’s multiple range test (*p <* 0.05).

The accumulation of K, Ca, Mg and Fe in leaves and roots of cucumber seedlings were affected by nitrate stress, MT and SNP treatments (Figure 2). Content of K, Mg and Fe were significantly reduced by HN treatment, but Fe content was increased in HN + SNP treated seedlings and decreased by HN + MT treatment, compared with HN treatment. Mg content in leaves and roots was decreased by HN + SNP treatment. The content of Ca was increased under nitrate stress, but it was significantly reduced by HN + MT and HN + SNP treatments in root, as compared with HN treatment.

### 2.2. Experiment II: Use of NO and MT to Reduce Nitrate Stress

#### 2.2.1. Growth Parameters, Pigments and Chlorophyll Fluorescence

**Figure 3:**
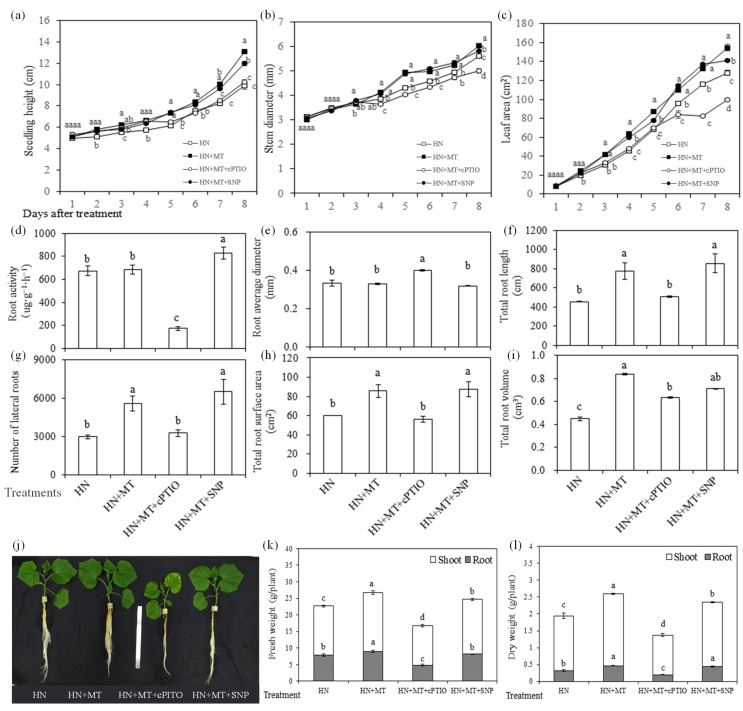
Effects of melatonin and nitric oxide treatments on growth parameters of cucumber seedlings under nitrate stress. (**a**) Changes of seedling height. (**b**) Changes of stem diameter. (**c**) Changes of leaf area. (**d**) Root activity. (**e**) Root average diameter. (**f**) Total root length. (**g**) Number of lateral roots. (**h**) Total root surface area. (**i**) Total root volume. (**j**) Appearance of seedlings. (**k**) Fresh weight. (**l**) Dry weight. Different letters indicate significant differences between treatments by Duncan’s multiple range test (*p <* 0.05).

The exogenous MT and NO on stem, diameter, leaf and root growth, as well as biomass accumulation were investigated in this study. As shown in Figure 3, with the growing of seedlings, parameters, such as height, stem diameter and leaf area, in the HN + MT and HN + MT + SNP treatment were significantly higher than those in HN and HN + MT + cPTIO from the 3rd to 5th days after treatment until the end of the experiment (Figure 3a–c). The fresh and dry weight of seedlings treated with HN + MT and HN + MT + SNP were greater than those of the seedlings treated with HN + MT + cPTIO and HN.

The root activity was significantly enhanced in the HN + MT + SNP treatment, which was significantly weakened by the HN + MT + cPTIO treatment, compared with HN and HN + MT (Figure 3d). Root average diameter in HN + MT + cPTIO was significantly higher than it in HN + MT treatments (Figure 3e). The number of lateral roots (Figure 3g), total root length (Figure 3f) and surface area (Figure 3h) were enhanced by the treatment of HN + MT and HN + MT + SNP, but they were greatly reduced in the presence of cPTIO. To compare with HN, the addition of MT enhanced total root volume of cucumber seedlings, and there was no significant difference between HN + MT and HN + MT + SNP (Figure 3i). Compared with HN + MT, root volume and surface area were not further enhanced by addition of SNP, indicated that endogenous was probably sufficient.

In accordance with parameters, visible leaf margin chlorosis was observed in cucumber seedlings treated with HN + MT + cPTIO during the experiment period (Figure 3j). Moreover, the enhanced biomass accumulation by addition of MT was reduced in the presence of cPTIO and SNP, the former was greater than the latter (Figure 3k,l).

**Figure 4:**
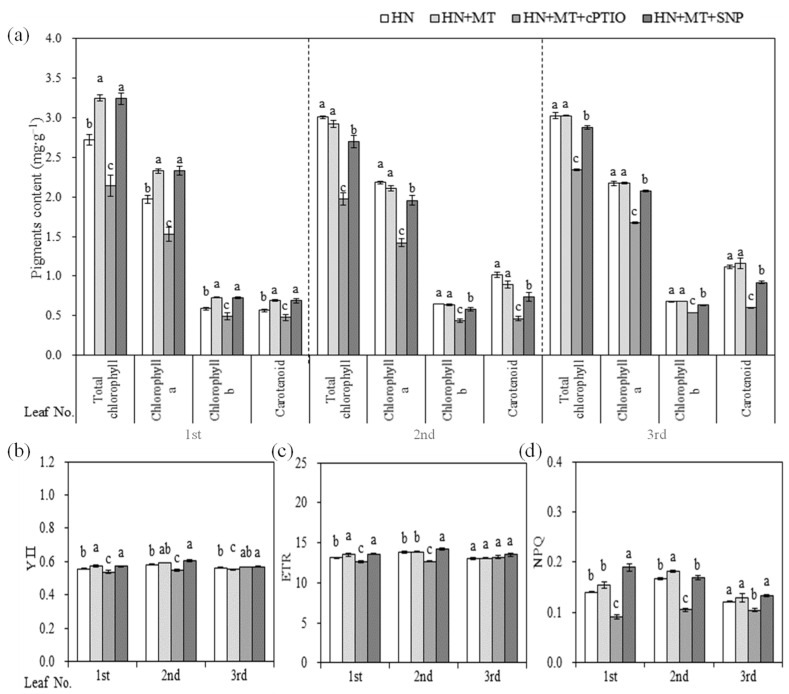
Effects of melatonin and nitric oxide on the pigments content and chlorophyll fluorescence characteristics of cucumber seedlings under nitrate stress. (**a**) Pigments content. (**b**) Effective quantum yield of photochemical energy conversion in PSII (YII). (**c**) Electron transport rate (ETR). (**d**) Non-photochemical quenching (NPQ). Error bars represent standard deviations of the means of three independent replicates. Different letters indicate significant differences between treatments by Duncan’s multiple range test (*p <* 0.05).

In the study here, pigments content and leaf chlorophyll fluorescence of the 1st, 2nd, 3rd true leaves were separately investigated. As shown in Figure 4, the differences of pigments content among treatments in the 1st leaf were different from that in the 2nd and 3rd leaf. Compared with HN, pigments content in the 1st leaf were significantly enhanced by the HN + MT and HN + MT + SNP treatments. In contrast, pigments content greatly decreased in the HN + MT + cPTIO treatment, as compared with in HN (Figure 4a). With the increase in leaf position, there was no significant difference between HN and HN + MT in the pigments content, HN + MT + SNP was lower than HN and HN + MT + cPTIO was the last. Differences of leaf chlorophyll fluorescence among treatments were indicated as greater in the 1st leaf than in the 2nd and 3rd leaves. Both YII and ETR of the 1st leaf were significantly improved in the HN + MT and HN + MT + SNP, but they were reduced in the presence of cPTIO. YII and ETR of three leaves in HN + MT + SNP were significantly increased, as compared with HN (Figure 4b,c). NPQ was increased by the addition of HN + MT + SNP and significantly reduced by the addition of cPTIO (Figure 4d). 

#### 2.2.2. Accumulation of Mineral Elements in Leaves and Root

**Figure 5:**
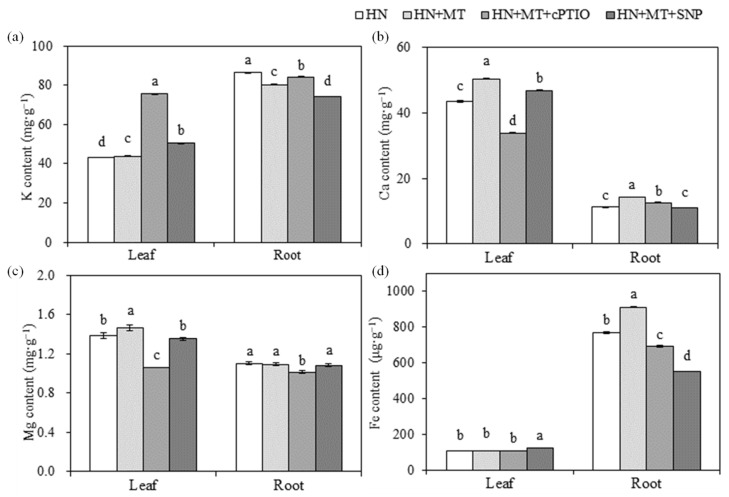
Effects of MT and NO on accumulation of mineral elements of cucumber seedlings under nitrate stress. (**a**) Potassium (K). (**b**) Calcium (Ca). (**c**) Magnesium (Mg). (**d**) Iron (Fe). Error bars represent standard deviations of the means of three independent replicates. Different letters indicate significant differences between treatments by Duncan’s multiple range test (*p* < 0.05).

As shown in Figure 5, K content in HN + MT + cPTIO were higher than in HN + MT (Figure 5a). The content of Ca, Mg and Fe in cucumber seedlings were higher in the HN + MT and HN + MT + SNP than in the HN, and Ca and Mg content in leaves of cucumber seedlings was significantly reduced in HN + MT + cPTIO treatment (Figure 5b–d). 

#### 2.2.3. Endogenous NO, MT, IAA and ABA Dynamic Change in the Root

**Figure 6:**
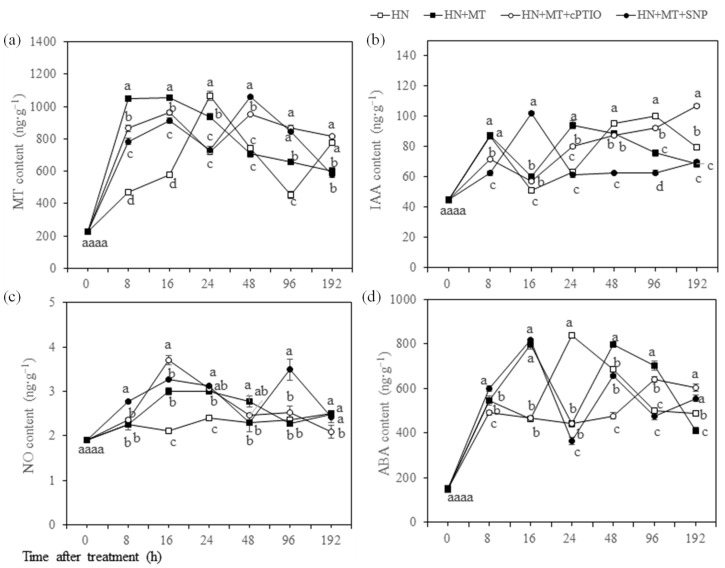
Effect of MT and NO on the endogenous content of MT (**a**), IAA (**b**), NO (**c**) and ABA (**d**) in the roots of cucumber seedlings under nitrate stress. Error bars represent standard deviations of the means of three independent replicates. Different letters indicate significant differences between treatments by Duncan’s multiple range test (*p* < 0.05).

The MT content in treatments of HN + MT, + SNP and + cPTIO were increased significantly within 16 h, as compared with HN. The MT content in HN + MT + SNP and HN + MT + cPTIO maintained a higher level than HN between 48 h and 96 h (Figure 6a). The IAA content reached up to the peak at 24 h after HN + MT treatment, and it was significantly higher than in the other treatments. IAA content in HN + MT + SNP treatment reached to peak at 16 h. In contrast, IAA content was significantly reduced at 96 h after treatments of HN + MT + SNP and HN + MT (Figure 6b). NO content tended to increase from 0 h to 16 h after treatments of HN + MT, + SNP and + cPTIO. NO content was significantly higher than in treatments of HN + MT, + SNP and + cPTIO than in HN treatment between 16 h and 24 h. NO was further increased in the HN + MT + SNP treatment at 8 h and 96 h. This indicated that exogenous MT-enhanced production of endogenous NO was associated with NO. The NO content increased to the highest level at 16 h after treatment of MT + cPTIO, but it reduced to the lowest level at 192 h (Figure 6c). Compared with HN + MT treatment, ABA content was significantly increased at 8 h after HN + MT + SNP treatment, but it was significantly decreased at 8 h after HN + MT + cPTIO treatment. ABA content significantly increased within 16 h in response to HN + MT and HN + MT + SNP treatment and reached to peak at 16 h. The ABA content maintained higher level between 48 and 96 h after HN + MT treatment than in HN + MT + SNP and HN (Figure 6d).

#### 2.2.4. NO, MT, IAA and ABA-Related Gene Expression Dynamics in the Root

**Figure 7:**
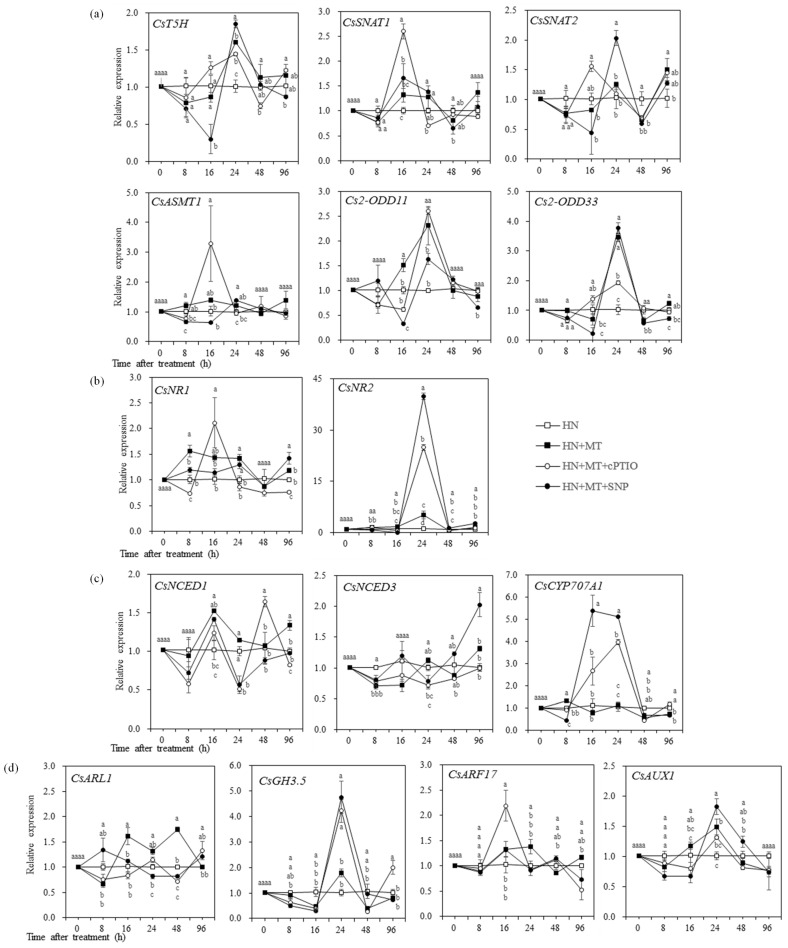
Effects of MT and NO treatments on expression of genes involved in MT metabolism (**a**), Nitrogen metabolism (**b**), ABA metabolism (**c**), Root growth and IAA transport (**d**) in roots of cucumber seedlings. Error bars represent standard deviations of the means of three independent replicates. Different letters indicate significant differences between treatments by Duncan’s multiple range test (*p* < 0.05).

The expression of NO, MT, IAA and ABA metabolism genes, as well as root growth related genes in the roots of cucumber seedlings were investigated in this study, including six genes involved in MT metabolism (*CsT5H*, *CsSNAT1*, *CsSNAT2*, *CsASMT1*, *Cs2-ODD11*, *s2-ODD33*), two genes involved in nitrogen metabolism (*CsNR1* and *CsNR2*), three genes involved in ABA metabolism (*CsNCED1*, *CsNCED*, *CsCYP707A1*) and four genes involved in IAA transport and root growth regulation (*CsARL*, *CsGH3.5*, *CsARF17*, *CsAUX1*). As shown in Figure 7a, the expression of *CsT5H* was significantly down-regulated at 16 h after HN–MT–SNP treatment, as compared with HN + MT. However, *CsT5H* was significantly upregulated at 24 h after treatments of HN + MT, + SNP and + cPTIO, as compared with HN, in which HN + MT + SNP was significantly higher than in HN + MT. The expression levels of *CsSNAT1* and *CsSNAT2* were significantly higher in HN + MT + SNP treatment than in HN + MT + cPTIO treatment at 24 h. Additionally, the expression of *Cs2-ODD11* and *Cs2-ODD33* was upregulated by HN + MT, compared with HN, and reached the peak at 24 h. The expression of *CsNR1* tended to increase within 24 h after HN + MT and HN + MT + SNP treatment, compared with HN. To compare with HN + MT, *CsNR1* was significantly upregulated at 96 h after HN + MT + SNP treatment. In contrast, it was down-regulated after HN + MT + cPTIO treatment through the whole period except 16 h, as compared with HN. The expression of *CsNR2* was significantly upregulated after the HN + MT + SNP treatment between 24 h and 96 h, as compared with HN + MT (Figure 7b). The expression of *CsNCED1* was significantly higher in HN + MT treatment than in HN treatment at 16 h and 96 h. The expression of *CsNCED3* at 96 h in HN + MT + SNP was significantly higher than it in HN + MT treatment. The expression of *CsCYP707A1* kept a higher level between 16 h to 24 h after HN + MT + SNP and HN + MT + cPTIO treatments than in HN and HN + MT (Figure 7c).

As shown in Figure 7d, the expression of *CsARL1* kept higher level in HN + MT than in HN between 16 h and 48 h after treatment. The expression of *CsGH3.5* in HN + MT + SNP and HN + MT + cPTIO treatments were significantly upregulated, as compared with HN + MT at 24 h. The expression of *ARF17* in HN + MT at 24 h was significantly upregulated, compared with the others. The expression of *ARF17* in HN + MT + cPTIO was significantly upregulated at 16 h, but down-regulated at 96 h, as compared with HN-MT. The expression of *CsAUX1* was significantly upregulated in HN + MT and HN + MT + SNP treatments at 24 h, compared with HN, and HN + MT + SNP was significantly higher than HN + MT.

## 3. Discussion

In general, both MT and NO have been reported to play an independently important role in plant stress responses [34]; NO evidently acts downstream of MT in promoting salt stress [35,36], sodic alkaline stress [25] and immune response [37], as well as adventitious root formation [30]. MT alleviate the inhibition of nitrate stress through modulating the nitrogen metabolism, two genes (*Cs-NR* and *Cs-GOGAT*) that function in nitrogen metabolism were significantly induced by MT [29]. However, the interaction of MT and NO on the nitrate stress tolerance of cucumber seedlings are not well understood. In the present study, in order to clarify the molecular mechanism of MT- and NO-induced nitrate tolerance, the photosynthesis capacity; pigment content; root morphological characteristics; accumulation of mineral elements; changes of endogenous NO, MT, IAA and ABA; and related gene expression were investigated in cucumber seedlings. The results showed that shoot height, stem diameter, leaf area, fresh weight and photosynthesis capacity of cucumber seedlings were significantly reduced under nitrate stress. However, the inhibition of growth and photosynthesis were independently mitigated by exogenous MT and NO (Table 1 and Figure 1).

Chlorophyll was easily destroyed during photosynthesis or under environmental stress [38]. MT could reduce the rate of chlorophyll degradation [39], and improve photosynthesis rates [15,29]. MT could also protect chloroplast membrane structure and grana lamellar structure of grape under drought stress [40]. In the present study, the content of Chla, Chlb and carotenoid in the 1st leaf of cucumber seedlings were significantly higher in HN + MT and HN + MT + SNP than in HN treatment (Figure 4a). The application of MT and SNP improved photosynthesis capacity (Figure 1b) and leaf Chl fluorescence (YII, ETR and NPQ (Figure 4b) under nitrate stress, but leaf pigments content and Chl fluorescence were significantly reduced in the presence of cPTIO (Figure 4a,b). This indicated that NO was essential for MT to enhance photosynthesis of cucumber seedlings grown under nitrate stress. Mg and Fe are important nutrient elements in photosynthesis, and other physiological and biochemical reactions related to plant growth and development; their deficiency often causes chlorosis and inhibits normal plant growth [41,42]. Excess nitrate was associated with a drop in levels of Mg in cucumber; MT promoted the accumulation of Mg under stress environmental [40]. Elevated MT levels induces NO accumulation; the NO signal upregulates the expression of iron-related genes, such as *FIT1*, *FRO2* and *IRT1*, thereby increasing the availability of soluble Fe [35]. In the present study, content of Fe in leaves and roots were significantly reduced by nitrate stress (Figure 2d), but it was significantly increased in HN + SNP (Figure 2d) and HN + MT (Figure 5d) treated roots of cucumber seedlings. Mg content in leaves of cucumber seedlings increased in HN + MT treatment (Figure 5c), and this promotion was weakened in the presence of cPTIO. Additionally, we observed obvious chlorosis in leaf margin (Figure 3j); this symptom was similar to Mg and Fe deficiency. These observations suggested that NO was required for MT to induce accumulation of Mg and Fe, thereby leading to an increase in photosynthesis capacity, greater leaf expansion, biomass accumulation and nitrate tolerance.

Previous research showed that MT plays a vital role in strengthening adventitious and lateral root formation and growth [23,30,33,43]. In the present study, the results showed that total root length, number of lateral roots and total root surface were higher in HN + MT and HN + MT + SNP treated cucumber seedlings than in HN + MT + cPTIO, and there was no significant difference between HN + MT and HN + MT + SNP (Figure 3f–h). This indicated that endogenous NO was required for MT to promote root elongation and to increase of the number of lateral roots of cucumber seedlings under nitrate stress. Similarly, previous research indicated that exogenous MT promoted the accumulation of endogenous NO; NO as a downstream signal, was involved in the MT-induced adventitious root formation [30]. The promotion of root growth may be the reason of the improved accumulation of Mg and Fe (Figure 5c,d), and it depended on endogenous NO content.

Ca provides intermolecular linkages and was considered to have a crucial function in stabilizing cell walls and membranes [44]. MT positively influences the integrity of cells and membranes by increasing the accumulation of Ca [29]. Ca level was significantly increased in plants treated with Ca(NO_3_)_2_ and KNO_3_ [29]. In the present study, Ca content in leaves was increased in HN + MT and HN + MT + SNP treated seedlings but reduced in cPTIO (Figure 5b). The Ca accumulation was consistent with enhanced root growth, indicating that Ca accumulation might be improved by strengthening root growth or that higher Ca accumulation promoted root growth by enhancing the stability of root cell walls and membranes; this will be discussed in further research. K is the most abundant cation in plants; biomass accumulation was significantly reduced in HN + MT + cPTIO treatments in the present study (Figure 3k,l); this is probably the reason of increased K content, but the accumulation amount in each plant was reduced (data not shown).

MT acts in a parallel way to IAA in both lateral and adventitious root induction [39]. Precious research showed that MT regulation of root morphogenesis was independent of IAA signal [45]. In transgenic plants that overproduce MT, a substantial decrease in IAA levels has been reported [13]. In the present research, endogenous MT content was enhanced by exogenous MT treatment within 24 h. In contrast, endogenous MT content was higher in HN + MT + cPTIO and HN + MT + SNP than in HN + MT between 48 h and 96 h (Figure 6a), suggesting that the enhancement was affected by endogenous NO content. In the present study, we found that the expression level of *Cs2-ODD11*, *Cs2-ODD33* and *CsT5H* were significantly higher in HN + MT treatment at 24 h, as compared with the HN, while *CsT5H* and *CsSNAT2* in HN + MT + SNP was significantly higher than in HN + MT at 24 h in HN + MT + SNP (Figure 7a). The induced genes of *CsT5H* and *CsSNAT2* at 24 h in the HN + MT + SNP treated seedlings coincided with the increasing of MT content at 48 h, suggesting that exogenous NO contributed to the MT induced of the MT synthesis gene expression, thereby promoting endogenous MT accumulation.

MT treatment induced a slight increase in endogenous IAA, compared with untreated plants, as has been observed in *Brassica juncea* [43] and tomato plants [30]. In the present study, the peak of IAA content in HN + MT + SNP and HN + MT treated seedlings successively appeared at 16 h and 24 h, and then decreased after 24 h (Figure 6a), suggesting that the promotion effects of MT and NO on IAA accumulation was within 24 h. Consistently, the expression of gene encoding auxin carriers (AUX1/LAX) was upregulated by HN + MT and HN + MT + SNP treatments at 24 h, and HN + MT + SNP was significantly higher than HN + MT (Figure 7d). It indicated that MT interacting with NO could improve IAA transport through regulating the transcription level of *CsAUX1*. These results indicated that exogenous MT alone or interacting with NO could promote root growth of cucumber seedlings by inducing accumulation of endogenous IAA and MT in roots, in which the improvement of endogenous MT was greater than IAA, the mechanism will be discussed in further research.

With regard to the accumulation of endogenous NO and expression of genes involved in nitrate synthesis, MT-induced the endogenous accumulation of NO, and it was further enhanced in the HN + MT + SNP treated seedlings at 8 h and 96 h (Figure 6c). Consistently, the expression of the *NR1* gene encoding nitrate reductase enzyme was induced by the treatment of HN + MT and HN + MT + SNP, but it was repressed in the presence of cPTIO under nitrate stress. *NR2* was significantly upregulated in the HN + MT + SNP- and HN + MT + cPTIO-treated seedlings, as compared with HN + MT (Figure 7b). It indicated that MT interacting with NO could enhance endogenous NO accumulation in cucumber seedling roots under nitrate stress, this depending on exogenous NO content.

The accumulation of ABA in plant cells is associated with the formation of ROS [46,47]. The application of MT results increased ABA content in drought-primed plants when exposed to cold stress [48]. MT treatments increased the levels of ABA and ethylene production and promoted berry ripening in a concentration-dependent manner by increasing endogenous MT content [48]. On the contrary, MT selectively down-regulates ABA synthesis gene *MdNCED3*, and upregulates ABA catabolic gene *MdCYP707A1* and *MdCYP707A2*, thereby reducing ABA contents in drought-stressed plants [23]. In the present study, *CsNCED1* was significantly upregulated by HN + MT and HN + MT + SNP treatments at 16 h (Figure 7c); this may be one of the reasons resulting in higher level of ABA content at 16 h under nitrate stress (Figure 6d). The treatments of HN + MT + SNP and HN + MT + cPTIO upregulated *CsCYP707A1* from 16 h and to 24 h, thereby resulting in low ABA content at 24 h, as compared with HN (Figure 6d). These results indicated that MT alone or MT interact with NO could increase endogenous ABA accumulation through regulating the genes of *CsNCED1* and *CsCYP707A1*; this depended on the NO content. The increasing accumulation of ABA indirectly indicated that the nitrate stress tolerance was improved by MT or MT + SNP under nitrate stress. Except for IAA and ABA, there are probably other hormones or substances associated with cell division that were regulated by MT and NO because the thickening of roots was greater than elongation in HN + MT + cPTIO treatment (Figure 3e), which would be discussed in our future research. There were about 320 genes related to rhizogenesis up and downregulated by MT in *Cucumber sativus* [33]. The expression levels of adventitious rootless 1 (*CsARL1*) can reflect adventitious root formation [49]. Auxin carriers (AUX1/LAX) regulates lateral root development, root gravitropism, root hairs and leaf phyllotaxy [50]. *CsARF17* is a negative regulator of adventitious root formation [51]. In the present study, the expression of *CsARL1* kept a higher level in HN + MT than in HN during 16 h and 48 h (Figure 7d). Similarly, expression of *CsAUX1* was significantly upregulated in HN + MT and HN + MT + SNP treatments at 24 h, compared with HN, and HN + MT + SNP was higher than HN + MT. The expression of *ARF17* in HN + MT + cPTIO was significantly upregulated at 16 h (Figure 7d). Generally, the upregulated genes of *CsARL1* and *CsAUX1* were closely related to enhanced root activity, root length and number of lateral roots (Figure 3f–h), as well as content of Ca, Mg and Fe in leaves (Figure 5b–d) of cucumber seedlings treated with HN + MT and HN + MT + SNP.

In the present research, we used SNP as NO donor and cPTIO as NO scavenger by referring to previous research [25,30,32]. However, the use of SNP was debatable in recent years [52] because it simultaneously releases NO, cyanide and iron in solution, cyanide and iron mask the NO effect of SNP, which probably limits its use as an NO donor. So, it is necessary to investigate the concentration of iron and cyanide in the medium of practice use in future research.

## 4. Materials and Methods

### 4.1. Plant Materials and Experimental Design

The two experiments were conducted in a greenhouse at College of Horticulture, South China Agricultural University. Germinated seeds of cucumber (*Cucumis sativus* L. cv. Jinyou No. 1) were sown in sponge blocks and irrigated with “Yamazaki” nutrient solution Ca(NO_3_)_2_·4H_2_O 826 mg·L^−1^, KNO_3_ 607 mg·L^−1^, NH_4_H_2_PO_4_ 153 mg·L^−1^, MgSO4·7H_2_O 370 mg·L^−1^, NaFe-EDTA 20 mg·L^−1^, H_3_BO_3_ 2.86 mg·L^−1^, MnSO_4_·4H_2_O 2.13 mg·L^−1^, ZnSO_4_·7H_2_O 0.22 mg·L^−1^, CuSO_4_·5H_2_O 0.08 mg·L^−1^, (NH_4_)_6_Mo_7_O_24_·4H_2_O 0.02 mg·L^−1^). Cucumber seedlings with one fully expanded true leaf were transplanted into hydroponic container. Aeration was switched on for 15 min every hour in hydroponic system. The average air temperature was 20 °C during greenhouse cultivation. After four days adaptation, nitrate nitrogen, MT, SNP, cPTIO were added into nutrient solution, constituting different treatment (Table 2), which were renewed every 3 days. 

There were four treatments in experiment one: (1) CK, cucumber seedlings were cultivated with “Yamazaki” nutrient solution, (2) HN, high nitrate nitrogen treatment, NO_3_^−^ concentration was upregulated to 75 mM in “Yamazaki” nutrient solution by increasing Ca(NO_3_)_2_ and KNO_3_, (3) HN + MT, high nitrate nitrogen treatment plus 2 μmol·L^−1^ melatonin (MT), (4) HN + SNP, high nitrate nitrogen treatment plus 100 μmol·L^−1^ sodium nitroprusside (SNP, a NO donor).

The second experiment included four treatments: (1) HN, high nitrate nitrogen treatment, NO_3_^−^ concentration was upregulated to 75 mM in Yamazaki nutrient solution by increasing Ca(NO_3_)_2_ and KNO_3_, (2) HN + MT, high nitrate nitrogen treatment plus 2 μmol·L^−1^ MT, (3) HN + MT + cPTIO, high nitrate nitrogen treatment plus 2 μmol·L^−1^ MT and 10 μmol·L^−1^ 2-(4-carboxyphenyl) -4, 4, 5, 5-tetramethylimidazoline-1-oxyl-3-oxide (cPTIO, a specific scavenger of NO), (4) HN + MT + SNP, high nitrate nitrogen treatment plus 2 μmol·L^−1^ MT and 100 μmol·L^−1^ SNP.

MT and cPTIO were produced by TCI, Co. Japan, SNP was produced by Damao Co., Tianjin, China. A total of 720 cucumber seedlings were cultivated in the second experiment. Three replicates for each treatment were adopted, and each replicate contained 30 seedlings. The uniform seedlings were randomly selected for parameters analysis after 16 days and 8 days treatment in experiment’s one and two, respectively.

### 4.2. Growth Parameters, Root Activity and Morphology Analysis

Shoot height of cucumber seedlings from stem basal to apical was measured by a ruler. Stem diameter below cotyledons was measured by a vernier caliper. Leaf area was measured by a leaf area meter (Li-3000A, LI-COR Inc., Lincoln, NE, USA). Seedlings were separated into shoot and root portions; fresh and dry weight were measured by electronic balance. Dry weight was determined after tissue was oven dried at 100 °C oven for 1 h and 75 °C oven for constant. The dried samples were used for analyzing the composition and content of mineral elements.

Lateral roots were firstly removed, then cultivated in different nutrient solution treatment for 8 days, then morphological parameters (number of lateral roots and root tips, root length, root diameter, root surface area and root volume) were measured by a root scanner (Perfection V700 Photo; EPSON, Nagano, Japan) and analyzed by using WinRHIZO 4.1 software (LC4800-II LA2400; Saint Foy, QC, Canada).

Root activity was determined by triphenyltetrazolium chloride (TTC) method [53] and expressed as the deoxidization ability. A total of 0.5 g of fresh root was immersed in 10 mL of equally mixed solution of 0.4% TTC and phosphate buffer (pH 7.0) and kept in the dark at 37 °C for 2 h. Then, 2 mL of 1 mol·L^−1^ H_2_SO_4_ was added to stop the reaction with the root. The root was dried with filter paper and then extracted with ethyl acetate. The extract was measured at 485 nm by a UV-spectrophotometer (UV-16A, SHIMADZU, Tokyo, Japan).

### 4.3. Photosynthesis and Chlorophyll Fluorescence Measurement

The net photosynthetic rates (Pn), stomatal conductance (Gs), intercellular CO_2_ concentration (Ci) and transpiration rate (Tr) was measured with a photosynthetic system (Li-6400, LICOR, Lincoln, NE, USA) on the second true leaf between 9:00 and 11:00 at 800 μmol·m^−2^·s^−1^. Chlorophyll and carotenoid content in the first, second and third true leaf were analyzed according to our previous research [54]. The sample (1.0 g) was homogenized by 20 mL of 80% (*v*/*v*) acetone. The absorbance at 663 nm, 645 nm and 440 nm were analyzed on a UV-spectrophotometer (UV-16A, SHIMADZU, Tokyo, Japan).

Chlorophyll fluorescence investigation was carried out in the first, second and third true leaf, which were dark adapted for 30 min prior to measurement. Measurements were performed by using a fluorescence imaging system-PAM (IMAG-MAXI; Heinz Walz, Effeltrich, Germany). The parameters of YII indicates the effective quantum yield of photochemical energy conversion in PSII, ETR represented electron transport rate through PSII, and the NPQ provides an estimate of the heat dissipation by the leaves.

### 4.4. Mineral Element Content Measurement

Cucumber seedlings were separated into shoot and root two portions and dried to constant weight at 75 °C after 1 h at 105 °C for quantification of potassium, calcium, magnesium and iron. A total of 0.3 g sample was mixed with 8 mL of concentrated H_2_SO_4_ in a digester tube, boiled gently until H_2_SO_4_ decomposing into a large amount of white smoke. Then, the temperature was increased until the solution became uniform brown and black. About 10 drops of H_2_O_2_ were added after for 5 min boiling; after cooling, we added 5 drops of H_2_O_2_ repeatedly, boiling again. This was repeated 3–5 times until the reaction solution become colorless or clear, then added water to a constant volume of 100 mL. The potassium was analyzed by use of flame atomic absorption spectrophotometer (Z-2300, Hitachi Instrument, Tokyo, Japan) equipped with potassium hollow cathode lamp, the presser of acetylene was adjusted to 0.085–0.090 kpa, the absorption wavelength was 248.3 nm.

A total of 2.0 g dried sample was mixed with 2.0 mL of 95% ethanol in a 30 mL porcelain crucible for pre-ash treatment. Reaction solution was heated in an electric furnace at 525 °C for 1 h, until the ash content was nearly white. Crucible was then removed to cool to room temperature and then moistened with a small amount of water, adding diluted hydrochloric acid solution to about 20 mL, boiled, then filtered and washed 3 times, dissolving the filtrate to constant volume at 100 mL. Then, the absorbance was read at 422.7 nm (calcium), 285.2 nm (magnesium) and 271.9 nm (iron) by use of flame atomic absorption spectrophotometer (FAAS, Z-2300, Hitachi Instrument, Tokyo, Japan) fit with hollow cathode lamp; the presser of acetylene was adjusted to 0.085–0.090 kpa.

### 4.5. Determination of Endogenous Content of IAA, ABA, MT and NO

Seedling root samples collected at 8 h, 16 h, 24 h, 48 h, 96 h and 192 h after treatments were rapidly frozen in liquid nitrogen and stored at −80 °C until use. Each treatment sample comprised three biological replicates. Each replicate was collected from at least six uniform seedlings. NO content was determined by using “NO kit”, (Shanghai Solarbio Biotechnology Co., Shanghai, China). The protein quantification was determined by BCA method, and diluted to 0.896 mg/mL, then determined the MT, IAA and ABA content. Contents of melatonin (MT), indole-3-acetic acid (IAA) and abscisic acid (ABA) were determined by using the “Plant hormone ABA ELISA kit” (MEIBIAO BIOLOGY, Nantong, China).

### 4.6. Quantitative Real-Time PCR Analysis

Seedling root samples collected at 8 h, 16 h, 24 h, 48 h and 96 h after treatment were rapidly frozen in liquid nitrogen and stored at −80 °C until use. Expression of genes involved in the biosynthesis or catabolism of NO, MT, IAA, ABA and root growth-related genes were evaluated by performing quantitative real-time PCR (qRT-PCR), as described in our previous research [55]. Total RNA was isolated from cucumber roots by using the RNAprep Pure Plant Kit (Tiangen Biotech Co. Ltd., Beijing, China). The RNA quality and purity were verified by Nanodrop 2000 and electrophoresis on 1.0% agarose gels. cDNA was synthesized from 1 μg of total RNA by using the PrimeScript TM RT Reagent Kit with gDNA Eraser (Perfect Real Time) (TaKaRa Bio, Inc., Shiga, Japan). Actin acted as the internal standard.

The qRT-PCR was conducted using the Light Cycler 480 II real-time PCR system (Roche, Basel, Switzerland) with one step SYBR Premix Ex Taq TM (Takara, Dalian, China). The reactions reagent mix was 5 μL SYBR Premix Ex Taq II, 1.0 μL cDNA template, 3.2 μL ddH_2_O, 400 nM of forward and 400 nM of reverse primer. The amplification program was 95 °C for 30 s and 40 cycles of 95 °C for 5 s and 60 °C for 30 s. Melting curve analyses were performed at the end of 40 cycles (95 °C for 5 s followed by a constant increase from 60 to 95 °C). Relative fold expression changes were calculated using the 2^−ΔΔCt^ method [56] with IQ5 software (BIO-RAD, Hercules, CA, USA). The qRT-PCR was performed in three technical replicates for each sample.

Genes were designed on the NCBI primer blast website (https://www.ncbi.nlm.nih.gov/tools/primer-blast/ accessed on 26 June 2022). The primer quality and efficiency were checked by qPCR on series of diluting cDNA. The primer sequences, Ct values and amplification efficiency and correlation coefficient for each gene are listed in the Supplementary Materials, Tables S1–S3.

### 4.7. Data Analysis

Significant differences among the treatments were determined by analysis of variance (ANOVA), followed by Duncan’s multiple range tests of SPSS 21.0 at *p* ≤ 0.05.

## 5. Conclusions

In our study, MT interacted with NO to mitigate nitrate stress by increasing the number of lateral root, root elongation, root activity, and accumulation of mineral elements (Ca, Mg and Fe), as well as the endogenous MT, NO, IAA, ABA in the roots cucumber seedlings. Correspondingly, the transcription levels of genes involved in the metabolism of nitrogen, IAA, ABA and MT (*CsNR1*, *CsNR2*, *CsAUX1*, *CsNCED3*, *CsT5H*) were induced by exogenous MT and NO under nitrate stress. This study provided deeper knowledge on the roles of MT in alleviating nitrate stress.

## Figures and Tables

**Table 1:** Effects of melatonin and nitrate oxide on growth parameters of cucumber seedlings grown under nitrate stress.

Treatment	Shoot Height	Stem Diameter (mm)	The 2nd No. Leaf Area (cm^2^)	The 4th No. Leaf Area (cm^2^)	Fresh Weight (g/Plant)	Dry Weight
	(cm)					(g/Plant)
CK	74.43 ± 1.15 a	7.24 ± 0.14 a	210.57 ± 18.31 a	233.10 ± 10.18 a	67.32 ± 0.72 a	4.99 ± 0.58 a
HN	49.45 ± 1.23 c	6.20 ± 0.03 c	116.49 ± 2.90 c	139.37 ± 5.45 bc	36.85 ± 1.67 d	2.96 ± 0.32 b
HN + MT	60.54 ± 1.56 b	6.70 ± 0.12 b	127.65 ± 4.96 c	131.68 ± 3.90 c	49.39 ± 1.00 c	4.42 ± 0.53 a
HN + SNP	62.36 ± 0.40 b	6.63 ± 0.01 b	152.75 ± 5.75 b	147.83 ± 2.35 b	54.96 ± 0.71 b	4.88 ± 0.38 a

Note: presented values are means ± SE. Different letters in the same column indicate significant differences (*p <* 0.05, *n* = 30).

**Table 2:** Nutrient solution treatments in two experiments.

Treatment		NO_3_	MT	SNP	cPTIO
		(mmol∙L^−1^)	(μmol∙L^−1^)	(μmol∙L^−1^)	(μmol∙L^−1^)
Expt. 1	CK	7	0	0	0
	HN	75	0	0	0
	HN + MT	75	2	0	0
	HN + SNP	75	0	100	0
Expt. 2	HN	75	0	0	0
	HN + MT	75	2	0	0
	HN + MT + cPTIO	75	2	0	10
	HN + MT + SNP	75	2	100	0

Note: MT, melatonin. SNP, sodium nitroprusside, a NO donor. cPTIO, 2,4-carboxyphenyl-4,4,5,5-tetramethylimidazoline-1-oxyl-3-oxide, a specific scavenger of NO.

## Data Availability

Not applicable.

## Supplementary Materials

The following supporting information can be downloaded at: https://www.mdpi.com/article/10.3390/molecules27185806/s1, Table S1: Sequences of primers used for this study. Table S2: Ct values for each gene involved in MT metabolism, nitrogen metabolism, ABA metabolism, root growth and IAA transport in roots of cucumber seedlings. Table S3: Amplification efficiency (E) and correlation coefficient (R^2^) for each gene.

## Sample Availability

Samples of the compounds are available from the authors.
